# microRNA-605 promotes cell proliferation, migration and invasion in non-small cell lung cancer by directly targeting LATS2

**DOI:** 10.3892/etm.2022.11415

**Published:** 2022-06-06

**Authors:** Ying Ye, Juhua Zhuang, Guoyu Wang, Saifei He, Jing Ni, Wei Xia, Jiening Wang

Exp Ther Med 14:867–873, 2017; DOI: 10.3892/etm.2017.4538

Following the publication of this paper, it was drawn to the Editors’ attention by a concerned reader that certain of the cell migration and invasion assay data shown in Fig. 5D were strikingly similar to data appearing in different form in other articles by different authors. Owing to the fact that the contentious data in the above article had already been published elsewhere, or were already under consideration for publication, prior to its submission to *Experimental and Therapeutic Medicine,* the Editor has decided that this paper should be retracted from the Journal. The authors were asked for an explanation to account for these concerns, but the Editorial Office did not receive a reply. The Editor apologizes to the readership for any inconvenience caused.

